# An Uncontracted Epstein-Nesbet Perturbation Theory Approximation to SC-NEVPT2 Based on Spin-Pure Selected CI Wave Functions

**DOI:** 10.1021/acs.jctc.6c00414

**Published:** 2026-07-08

**Authors:** Mihkel Ugandi, Michael Roemelt

**Affiliations:** Institut für Chemie, Humboldt-Universität zu Berlin, Brook-Taylor-Str. 2, Adlershof, 12489 Berlin, Germany

## Abstract

Recently, we have developed a spin-adapted selected configuration interaction (SCI) method for capturing static electron correlation effects in large active orbital spaces and the corresponding nuclear gradients for geometry optimizations. This work reports two methods to incorporate dynamical electron correlation effects on top of the SCI wave function. First, we implemented the strongly contracted variant of *N*-electron valence state perturbation theory (SC-NEVPT2) including the residual terms emerging from the incompleteness of the SCI wave function. Through utilization of prototyping symmetries, efficient selection procedures and specifically designed parallelization/batching schemes, the steeply scaling computational costs and memory requirements could be alleviated. Second, we developed an approach where the notoriously problematic ${\mathrm{V̂}}_{a}^{-1}$ and ${\mathrm{V̂}}_{i}^{+1}$ perturber classes are treated by Epstein–Nesbet (EN) perturbation theory. The resulting hybrid EN-SC-NEVPT2 method is able to tackle active spaces of up to 30 orbitals with significantly lower computational costs than SC-NEVPT2. A set of test calculations illustrate the performance of the implemented methods.

## Graphical abstract

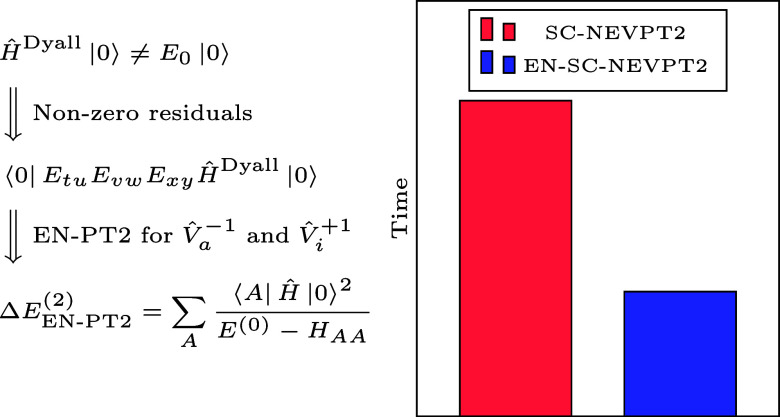

## Introduction

Multireference (MR) quantum chemistry methods are well established for carrying out calculations on molecular systems with nearly degenerate electronic configurations. Such degeneracies commonly occur during homolytic bond breaking processes, in transition metal complexes and in electronically excited states.^1–8^ The resulting multiconfigurational character of the electronic states typically involves only a comparably small subset of orbitals. Conventionally, this space of so-called active orbitals has been treated at the level of full configuration interaction (FCI), yielding the complete active space (CAS) method.^9,10^ The CAS approach recovers all correlation effects within the space of active orbitals, but unfortunately, scales exponentially with increasing active space size. At the same time it is well-known that FCI in many cases incorporates many “deadwood” configurations that do not contribute significantly to the state of interest.^11,12^ Therefore, a lot of effort has been invested over the last three decades into developing approximate active space methods. Prominent examples of such methods are based on the density matrix renormalization group (DMRG)^13–15^ or quantum Monte Carlo (QMC) algorithms.^16,17^ In addition, the selected configuration interaction (SCI) Ansatz has gained increasing popularity particularly in the more recent years.^4,18–20^ Among the SCI approaches, the heatbath-CI method first introduced by Holmes et al.,^21^ is particulary efficient.^22^ In the SCI domain, we have contributed the configuration-based heat-bath CI (CFG-HCI) method that serves as a cornerstone for the dynamical electron correlation methods developed in this work.^1^

Although aforementioned approximate FCI solvers enable calculating nearly exact correlation energies for smaller molecules, they are inherently inefficient for capturing dynamical electron correlation effects in large molecules. On the other hand, perturbation theory and coupled cluster approaches have been established as tools for an effective treatment in the dynamical electron correlation regime where the wave function has predominantly single reference character.^23^ Therefore, it is common to combine approaches from both realms to obtain a realistic description of multiconfigurational states in large molecules: Static correlation effects are accounted for using an exact or approximate CAS approach, while the remaining dynamical correlation is incorporated using, e.g. perturbation theory. This was done, for example, in the seminal work by Andersson et al. developing the complete active space perturbation theory (CASPT2) method.^24,25^ In its original formulation the CASPT2 Ansatz suffers from the occurrence of so-called intruder states prompting the development of strategies to avoid this behavior, mostly through usage of empirical shift parameters.^26,26,27^ An alternative approach based on second order perturbation theory was introduced by Angeli et al. in the early two thousands with the development of *n*-electron valence state perturbation theory (NEVPT2).^3,28^ By making use of the Dyall Hamiltonian, the NEVPT2 method is resilient to the occurrence of intruder states and does not require using an empirical level shift parameter.^29^ Both CASPT2 and NEVPT2 have been implemented some time ago for DMRG and QMC references thus allowing for their application for fairly large active spaces.^30–34^ Recently, Min and Park also implemented CASPT2 and NEVPT2 methods based on the ASCI wave function Ansatz for the active space.^35^ However, none of these implementations take into account the incomplete nature of the underlying DMRG, QMC and adaptive sampling CI (ASCI) wave functions. Specifically, it was later demonstrated how using an approximate CAS wave function gives rise to the so-called residual terms.^36,37^ Only very recently, Guo et al. incorporated such terms in their combination of NEVPT2 and the iterative-Configuration expansion CI (ICE-CI).^38^ Both recent works on PT2 methods with selected CI reference functions presented impressive calculations featuring large active spaces with up to 34 electrons in 34 orbitals which demonstrates the enormous potential of this combination. At the same time, the largest calculations were enabled through usage of “aggressive truncation parameters” (Guo et al.^38^) and “quite small” number of selected determinants (Min et al.^35^) thus highlighting the great algorithmic challenges faced while formulating efficient and accurate PT2 methods based on selected CI references.

In the present work, we report an implementation of the strongly contracted variant of NEVPT2 (SC-NEVPT2) based on our recently introduced selected CI approach.^1,2^ Importantly, the presented method incorporates aforementioned residual terms. A high level of efficiency is reached through usage of efficient screening techniques evolved from the heatbath-CI rationale,^21^ exploitation of prototyping symmetry^4,39^ and parallelization with the prefix-algorithm.^1^ Additionally, an alternative approach is presented where the expensive terms originating from the ${\mathrm{V̂}}_{a}^{-1}$ and ${\mathrm{V̂}}_{i}^{+1}$ perturber classes are treated using an uncontracted second-order Epstein-Nesbet perturbation theory approach (EN-SC-NEVPT2). In the following, the basics of CFG-HCI are reviewed along with a description of the SC-NEVPT2 and EN-SC-NEVPT2 implementations. The performance of the developed methods is demonstrated through their application to a range of molecular systems.

## Theory and Implementation

In the following, the indices *i*, *j*, *k*, *l* are used for the internal molecular orbitals; a, b, c, d for externals; t, u, v, w, x, y for active orbitals; and p, q, r, s for general orbitals. The capital indices I and J refer to occupation number vectors (ONV) or orbital configurations (CFG), whereas the Greek letters, μ and ν, are used to indicate the spin-coupling. Thus, we denote configuration state functions (CSF) as |*I*μ⟩ −when the Greek letter is omitted, the spin-couplings are assumed implicitly. As usual, we define the second-quantized molecular Hamiltonian as

1 $$Ĥ=\sum_{pq}h_{pq}E_{pq}+\frac{1}{2}\sum_{pqrs}\left(pq|rs\right)e_{pq,rs}$$

with *h*_pq_ and (*pq*|*rs*) being the one-and two electron integrals. The two-electron excitation operator, *e*_pq,rs_ = *E*_pq_ *E*_rs_ – δ_rq_ *E*_ps_, is defined in terms of the spin-traced one-electron excitation operator *E*_pq_ = *a*_pα_^†^ *a*_qα_+*a*_pβ_^†^ *a*_qβ_. The Dyall Hamiltonian used in this work is defined as^28,29^

2 $$\begin{array}{ll} {Ĥ}^{D} & ={Ĥ}_{act}^{D}+{Ĥ}_{inact}^{D}\\ {Ĥ}_{act}^{D} & =\sum_{tu}F_{tu}^{C}E_{tu}+\frac{1}{2}\sum_{tuvw}\left(tu|vw\right)e_{tu,vw}\\ {Ĥ}_{inact}^{D} & =\sum_{i}\varepsilon_{i}E_{ii}+\varepsilon_{a}E_{aa}+C \end{array}$$

with some additional quantities: The core Fockian – $F_{pq}^{C}=h_{pq}+\sum_{i}\left[2\left(ii|pq\right)-\left(ip|iq\right)\right]$ ; constant *C* = 2∑_*i*_ *h*_*ii*_ + ∑_*ij*_[2­(*ii*|*jj*) – (*ij*|*ij*)] – 2∑_*i*_ε_*i*_; and the orbital energies ε_*i*_ = *F*_*ij*_δ_*ij*_ and ε_*a*_ = *F*_ab_δ_ab_, that are obtained by diagonalizing the generalized MCSCF Fockian in the internal and external orbital spaces.

### Configuration-Based HCI

The precursor to this work was the development of a spin-pure selected CI method that utilizes orbital configurations (CFGs) as building blocks for the many-body wave function basis,^1^ i.e. the wave function is expanded in CSFs that are organized in complete sets belonging to individual CFGs. The corresponding ground state wave function and the energy eigenvalue equation may be written as

$$|0\rangle =\underset{I\in V_{0}}{\sum }\underset{\mu \in I}{\sum }C_{I\mu }|I\mu \rangle ,\mathrm{and},{\mathrm{P̂}}_{0}Ĥ{\mathrm{P̂}}_{0}|0\rangle =E^{\left(0\right)}|0\rangle$$ 3

where ${\mathrm{P̂}}_{0}$ is the projection onto the space of selected CFGs (CSFs). A prominent feature of the CFG ansatz is the fact that for each CFG all spin-couplings are taken into account. However, the explicit knowledge of the spin-couplings is necessary only for the calculation of the electronic coupling coefficients^39^

4 $$A_{tu}^{I\mu ,J\nu }=\langle I\mu |E_{tu}|J\nu \rangle$$

The benefits of this approach are (1) precalculation of a limited set of nonredundant electronic coupling coefficients; (2) efficient implementation owing to the high degree of vectorization. As a downside, the wave function is less compact because some of the spin-couplings may not be important for an accurate description of the wave function.^4,40^

A key ingredient to the efficient configuration selection in our implementation is the heat-bath CI scheme put forth by Holmes et al.^21^ Therefore, we shall refer to the discussed implementation as CFG-HCI. In CFG-HCI, the selection is carried out in two steps. First, a candidate set of CFGs is created using the HCI screening on configurations obtained via one-and two-electron excitations on the so-called generator CFGs. The generators are defined as a subset of the current variational space CFGs^4^

5 $$V_{G}=\left\{|I\rangle \in V_{0}|\max_{\mu }\left|C_{I\mu }\right|>T_{gen}\right\}$$

The HCI screening uses importance functions defined on the candidate CFGs for the one-and two-electron excitations

6 $$|A\rangle =E_{pq}|I\rangle \to f_{HCI}^{1}\left(|A\rangle \right)=\max_{\mu }\left|h_{pq}\cdot C_{I\mu }\right|$$

7 $$|A\rangle =E_{pq}E_{rs}|I\rangle \to f_{HCI}^{2}\left(|A\rangle \right)=\max_{\mu }\left|g_{pq,rs}\cdot C_{I\mu }\right|$$

Both importance functions are compared against the same threshold, *T*_var_. The efficiency of the HCI scheme originates from sorting the integrals and CI coefficients beforehand so that the loops creating excited CFGs can be terminated early. Doing so, the majority of the unimportant CFGs will not be considered.^21^ In a second step, all CFGs selected by HCI are pruned using the more rigorous CIPSI criterion^41,42^

8 $$f_{CIPSI}\left(|A\rangle \right)=\max_{\mu }\left|\frac{\sum_{I\nu }H_{A\mu ,I\nu }C_{I\nu }}{E^{\left(0\right)}-H_{A\mu ,A\mu }}\right|>T_{var}$$

The last step is important as the presence of the energy denominators in CIPSI yields significantly more compact wave functions as HCI alone.^19^ Using these two selection methods, the wave function is expanded iteratively until (1) no new CFGs are found; (2) the energy difference between two steps becomes sufficiently small (by default 10^–5^ Eh).

As common in SCI methods, the CFG-HCI method can calculate a final, albeit optional, second-order perturbation theory (PT2) energy correction within the active space

9 $$\Delta E_{PT2}=\sum_{A\mu }\frac{\langle A\mu |Ĥ|{0\rangle }^{2}}{E^{\left(0\right)}-H_{A\mu ,A\mu }}$$

This correction is particularly important when high accuracy is pursued, as for example, in benchmark calculations.^43^ In practice, the space of singly and doubly excited CFGs can become enormous and require too large amounts of computer memory.^44^ In the next section we describe an algorithm that enables calculating the CFG-HCI PT2 correction on-the-fly without exceeding available memory. This algorithm is also crucial to the memory-efficient calculation of certain quantities required by the NEVPT2 methods implemented in the present work.

### CFG-Prefix Algorithm

The generation of singly and doubly excited configurations poses certain technical challenges. Orbital CFGs bring the advantage of compact representation of the wave function because the spin-couplings need not be explicitly assigned to each occupation number vector (ONV). Nevertheless, in calculations with very large active spaces the memory requirement to store all excited CFGs can become substantial. This issue becomes more prominent when one is trying to create the excited CFGs in parallel. To elaborate on this further, consider these naive algorithmic steps to create singly excited CFGs:

1. Loop over all reference space CFGs, |*I*⟩ ∈ *V*_0_.
2. Loop over all possible *p*, *q*-indices and carry out *E*_*pq*_|Φ⟩ = |Φ′⟩ if possible.

A naive strategy here would be to split the first loop among parallel threads in a round-robin fashion. The first problem that would occur is that different threads might generate overlapping sets of excited CFGs. This would result in an even larger memory requirement since the threads should store all their generated CFGs before parallel reduction occurs. Second, on-the-fly parallel reduction would cause large synchronization overhead while at-the-end reduction would be time-consuming due to merging large quantities of redundant data. Lastly, this redundancy in the overlapping CFGs among threads can be expected to increase with the number of threads. We believe that these arguments render the naive strategy poorly scalable.

A more suitable approach to the parallel and/or batchwise computation of the perturbative correction in eq 9 and other similar quantities (see below) is to split and distribute the space of excited configurations {|*A*⟩}. However, a naive division of all singly and doubly excited configurations would entail the generation of the entire space at high costs. To overcome this problem, we have developed an algorithm based on configuration prefixes. Essentially, a CFG prefix is a segment of the ONV with the length smaller than the number of orbitals. For instance, we may create a CFG prefix with the length *P* as

10 $$|I\rangle =|n_{1}^{I},...,n_{P}^{I},...,n_{K}^{I}\rangle \to |I_{P}\rangle =|n_{1}^{I},...,n_{P}^{I}\rangle$$

The idea of the prefix algorithm is to divide the space of excited configurations based on their occupation patterns within the prefix. To this end, we split the excitation process on an arbitrary reference CFG such that part of it gets carried out on the prefix beforehand, and the remaining part on the excited CFG-prefix when the excited CFG is actually used. Depending on the chosen prefix-length, the space of excited prefixes can be made much smaller than that of the excited CFGs. Hence, the excited prefixes can be generated fast beforehand, and by a single thread. In our implementation, the prefix size is chosen to be the length of the doubly and singly occupied part of the ROHF CFG. It is important to emphasize that only single and double excitations need to be considered in order to create the prefixes.

To conclude this part, consider an example algorithm for calculating the core part of the three-particle density matrix (3PDM) which is defined as

11 $$\Gamma_{tuvwxy}^{core}=\langle 0|E_{tu}E_{vw}E_{xy}|0\rangle$$

we plug in the RI expansion yielding one-and two-electron connections from the reference to RI CFGs

12 $$\Gamma_{tuvwxy}^{core}=\sum_{K}\langle 0|E_{tu}E_{vw}|K\rangle \langle K|E_{xy}|0\rangle$$

In calculating the core-3PDM, only singly excited prefixes |*K*⟩ are created from |0⟩. The algorithm is summarized in Algorithm 1. We note that the required one-electron coupling coefficients, defined in eq 4, are calculated beforehand using a similar, but simpler algorithm. The two-electron coupling coefficients, defined here as *B*_tu,vw_^*IμJν*^ = ⟨*I*μ|*E*_tu_ *E*_vw_|*Jν*⟩, are calculated on the fly via matrix mulciplication

13 $$B_{tu,vw}^{IJ}=A_{tu}^{IK}A_{vw}^{KJ}$$

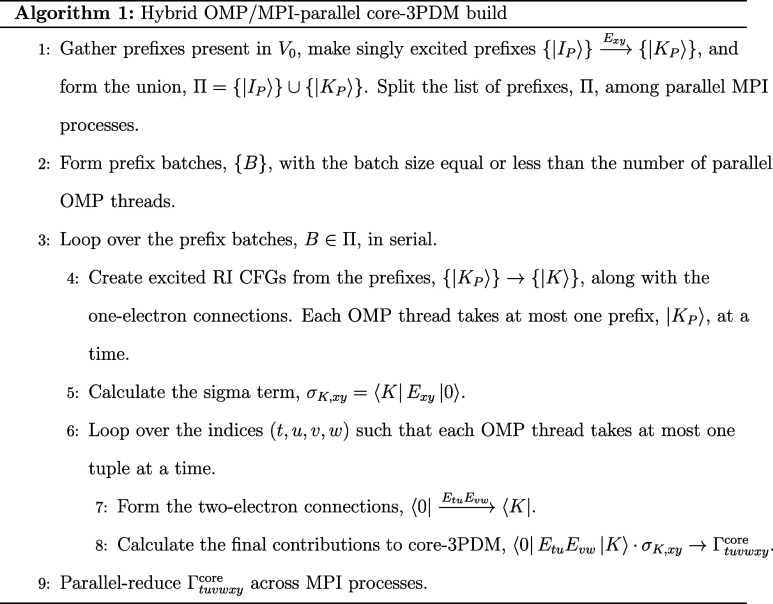

### SC-NEVPT2

In standard Rayleigh–Schrödinger perturbation theory (RSPT), the Hamiltonian is partitioned as

14 $$Ĥ={Ĥ}_{0}+\mathrm{V̂},with,\mathrm{V̂}=Ĥ-{Ĥ}_{0}$$

The second order energy correction to the zeroth order energy can be calculated from

15 $$E^{\left(2\right)}=\langle 0^{\left(0\right)}|\mathrm{V̂}|0^{\left(1\right)}\rangle$$

where the first order wave function is determined by the equation^23^

16 $$\left({Ĥ}_{0}-E^{\left(0\right)}\right)|0^{\left(1\right)}\rangle =-\left(\mathrm{V̂}-E^{\left(1\right)}\right)|0^{\left(0\right)}\rangle$$

By making an expansion for the first order wave function correction

17 $$|0^{\left(1\right)}\rangle =\sum_{A}T_{A}^{\left(1\right)}|A\rangle$$

the expansion amplitudes can be calculated from

18 $$\sum_{A}\langle B|{Ĥ}_{0}-E^{\left(0\right)}|A\rangle T_{A}^{\left(1\right)}=-\langle B|\mathrm{V̂}|0^{\left(0\right)}\rangle$$

assuming that the perturbers, |*A*⟩, are orthogonal to the reference, |0^(0)^⟩. However, the zeroth order Hamiltonian is not necessarily diagonal in the space of perturbers. This would warrant a costly iterative solution to the problem at hand. First, in *N*-electron valence state perturbation theory (NEVPT), the zeroth order Hamiltonian is chosen to be the Dyall Hamiltonian.^28,29^ This choice has been particularly successful in making the NEVPT approach resilient to the occurrence of intruder states. Further simplifications to solving eq 18 are typically made by introducing the contraction approximation.^3,28^ Thus, both the partially and strongly contracted variants are in practical use. They differ by the extent to which contractions are carried out in defining the perturbers, |*A*⟩. A particularly convenient option is the strongly contracted (SC−) variant where the perturbers are chosen such that the zeroth order Hamiltonian is diagonal in that basis. This enables a noniterative calculation of the second order energy correction, reminiscent of the single reference second-order Møller–Plesset theory. Thus, we employ the SC-NEVPT2 variant for the developments in this work.

For explicit expressions of the strongly contracted perturbers, we refer the reader to the original work by Angeli et al.^3^ In here, we shall adopt the convention to write the perturbers as

19 $$|\Phi_{l}^{\left(k\right)}\rangle ={\mathrm{V̂}}_{l}^{\left(k\right)}|0^{\left(0\right)}\rangle$$

where (*k*) counts the total balance of moved electrons in the active space. The subscript *l* refers to the set of internal and external orbital indices involved in the excitation facilitated by ${\mathrm{V̂}}_{l}^{\left(k\right)}$ . According to their excitation pattern, the perturber functions that are part of the first order interacting space can be divided in eight different classes, i.e {Φ_*ijab*_^(0)^}, {Φ_*iab*_^(−1)^}, {Φ_*ija*_^(1)^}, {Φ_*ab*_^(−2)^}, {Φ_*ia*_^(0)^}, {Φ_*ij*_^(2)^}, {Φ_*a*_^(−1)^} and {Φ_*i*_^(1)^}. In SC-NEVPT2, the second order energy correction is calculated from^28^

20 $$E^{\left(2\right)}=\sum_{kl}\frac{N_{l}^{\left(k\right)}}{E^{\left(0\right)}-E_{l}^{\left(k\right)}}$$

where *E* ^(0)^ refers to the reference energy; *N*_*l*_^(*k*)^ and *E*_*l*_^(*k*)^ refer to the perturber norms and energies, respectively. The zeroth-order energy is obtained by diagonalizing the Hamiltonian in a reference space, as in eq 3. The perturber norms and energies are defined as

21 $$N_{l}^{\left(k\right)}=\langle 0|{\left({\mathrm{V̂}}_{l}^{\left(k\right)}\right)}^{†}{\mathrm{V̂}}_{l}^{\left(k\right)}|0\rangle$$

22 $$E_{l}^{\left(k\right)}=\frac{1}{N_{l}^{\left(k\right)}}\langle \Psi_{l}^{\left(k\right)}|{Ĥ}^{D}|\Psi_{l}^{\left(k\right)}\rangle$$

where the Dyall Hamiltonian was used in the last expression. Following Angeli et al., we write the perturber energies as^3^

23 $$E_{l}^{\left(k\right)}=\frac{1}{N_{l}^{\left(k\right)}}\langle 0|{\left({\mathrm{V̂}}_{l}^{\left(k\right)}\right)}^{†}{\mathrm{V̂}}_{l}^{\left(k\right)}{Ĥ}^{D}|0\rangle +\frac{1}{N_{l}^{\left(k\right)}}\langle 0|{\left({\mathrm{V̂}}_{l}^{\left(k\right)}\right)}^{†}\left[{Ĥ}^{D},V_{l}^{\left(k\right)}\right]|0\rangle$$

This further treatment brings certain advantages in evaluating the perturber energies. The second term on the right-hand side now involves a commutator with the Dyall Hamiltonian, leading to a rank reduction of the excitation operators. In the first term, the Dyall Hamiltonian picks up the energy in case of complete active spaces. For incomplete active spaces considered here, this is not the case however. Nevertheless, it will be shown later that a convenient intermediate can be introduced.

We proceed, by writing the rightmost term as

24 $$\frac{1}{N_{l}^{\left(k\right)}}\langle 0|{\left({\mathrm{V̂}}_{l}^{\left(k\right)}\right)}^{†}\left[{Ĥ}^{D},{\mathrm{V̂}}_{l}^{\left(k\right)}\right]|0\rangle =\Delta \varepsilon_{l}+\frac{1}{N_{l}^{\left(k\right)}}\langle 0|{\left({\mathrm{V̂}}_{l}^{\left(k\right)}\right)}^{†}\left[{Ĥ}_{act}^{D},{\mathrm{V̂}}_{l}^{\left(k\right)}\right]|0\rangle$$

where Δε_*l*_ is the sum of virtual orbital energies minus the internal ones. The first term on the right-hand side in eq 23 requires special attention.^32,36–38,45^ Due to the fact that |0⟩ is not an eigenfunction of the Dyall Hamiltonian, we define a residual term as

25 $$R_{l}^{\left(k\right)}=E^{\left(0\right)}-\frac{1}{N_{l}^{\left(k\right)}}\langle 0|{\left({\mathrm{V̂}}_{l}^{\left(k\right)}\right)}^{†}{\mathrm{V̂}}_{l}^{\left(k\right)}{Ĥ}^{D}|0\rangle$$

that vanishes for CAS wave functions, but not in case of approximate CAS. Before discussing the treatment of this term for SCI wave functions, let us finalize the second-order energy expression for SC-NEVPT2 with the residual terms. Defining the commutator term

26 $$O_{l}^{\left(k\right)}=\langle 0|{\left({\mathrm{V̂}}_{l}^{\left(k\right)}\right)}^{†}\left[{\mathrm{V̂}}_{l}^{\left(k\right)},{Ĥ}_{act}^{D}\right]|0\rangle$$

the second-order energy correction is written as

27 $$E^{\left(2\right)}=\sum_{kl}\frac{N_{l}^{\left(k\right)}}{R_{l}^{\left(k\right)}-\Delta \varepsilon_{l}-O_{l}^{\left(k\right)}/N_{l}^{\left(k\right)}}$$

The explicit expressions for the norms and commutator terms are given in the original work by Angeli et al.^3^ In this work, we provide additional expressions for the residuals that can be found in the Supporting Information.

It has been noted that neglect of the residuals defined in eq 25, leads to the so-called false intruder states and consequently unreliable results.^36^ For a stable dynamical correlation calculation, it is necessary to incorporate these terms. Early in the course of this work, we realized that the residuals can be conveniently obtained from the perturber norms defined in eq 21 by plugging in the Dyall Hamiltonian and employing the resolution of identity (RI) in the CSF-space

28 $$D_{l}^{\left(k\right)}=\langle 0|{\left({\mathrm{V̂}}_{l}^{\left(k\right)}\right)}^{†}{\mathrm{V̂}}_{l}^{\left(k\right)}{Ĥ}^{D}|0\rangle =\sum_{K}\langle 0|{\left({\mathrm{V̂}}_{l}^{\left(k\right)}\right)}^{†}{\mathrm{V̂}}_{l}^{\left(k\right)}|K\rangle \langle K|{Ĥ}^{D}|0\rangle$$

The Dyall Hamiltonian connects the CFGs |*K*⟩ to the reference wave function through active space orbital excitations. Thus, the RI space CFGs have the same occupation pattern as the zeroth order approximate CAS basis functions. We note that this space is generally larger than that of spanned by the reference CFGs, $|I\rangle \in V_{0}$ . This is so because the active part of the Dyall Hamiltonian may generate CFGs not already present in |0⟩. Nevertheless, the RI CFGs possess the same occupational patterns as the zeroth order CFGs: doubly occupied and empty external orbitals; and active orbitals with varying occupation. Therefore, we may use the same expressions for the residuals as for the norms, except the PDMs will have to be replaced with a corresponding expression that involves the Dyall Hamiltonian. For more details and the explicit expressions, please refer to the Supporting Information. An important aspect that we would like to highlight here is the occurrence of up to 5-electron excitation operator matrix elements. Specifically, the new quantities to be calculated are

29 $$\Delta_{tu}^{1}=\langle 0|E_{tu}{Ĥ}_{act}^{D}|0\rangle$$

30 $$\Delta_{tuvw}^{2}=\langle 0|E_{tu}E_{vw}{Ĥ}_{act}^{D}|0\rangle$$

31 $$\Delta_{tuvwxy}^{3}=\langle 0|E_{tu}E_{vw}E_{xy}{Ĥ}_{act}^{D}|0\rangle$$

in the presented implementation these Dyall tensors are calculated using the configurational resolution of identity.^45^ However, that alone is not sufficient due to large computer time and memory demands.^38^ To reduce calculation times, we apply a screening in evaluation of the Δ^3^ term

32 $$\left|C_{I}\right|\cdot \left|\sigma_{K}\right|<T^{D^{3}}$$

here, *I* refers to a reference space CFG that connects to the RI space CFG *K* through three-electron connections. The sigma-vector-like term is defined as

33 $$\sigma_{K}=\langle K|{Ĥ}_{D}|0\rangle$$

in large active space calculations, the space of RI configurations can become too large to be stored in memory. The *K*-configurations are thus handled on-the-fly using the configuration-prefix algorithm proposed in the original CFG-HCI method.^1^ We conclude by pointing the reader to the Supporting Information for the specific algorithms involved in the presented HCI-SC-NEVPT2 implementation.

### EN-PT2

The zeroth order Hamiltonian in Epstein-Nesbet perturbation theory (EN-PT) is defined as^46–48^

34 $${Ĥ}_{EN‐PT2}=\sum_{IJ\in V_{0}}\langle I|Ĥ|J\rangle |I\rangle \langle J|+\sum_{A\in V^{\prime }}\langle A|Ĥ|A\rangle |A\rangle \langle A|$$

The first summation runs over CSFs in the variational space whereas the second summation runs over CSFs that belong to the orthogonal perturber space. With such a zeroth order Hamiltonian, the second-order energy correction (EN-PT2) is calculated as

35 $$\Delta E^{EN‐PT2}=\sum_{A\in V^{\prime }}\frac{{\left(\sum_{I\in V_{0}}\left\langle A\right|Ĥ|I\rangle C_{I}\right)}^{2}}{E^{\left(0\right)}-\langle A|Ĥ|A\rangle }$$

Commonly, this perturbative correction is employed in SCI methods to improve the active space energy.^22,44,49^ In this work, we mix the zeroth order Hamiltonian with EN-PT theory to calculate the energy correction within the ${\mathrm{V̂}}_{a}^{\left(-1\right)}$ and ${\mathrm{V̂}}_{i}^{\left(+1\right)}$ perturber spaces of SC-NEVPT2.^50^ Thus, the working zeroth order Hamiltonian used here can be written as

36 $${Ĥ}_{0}={Ĥ}_{D}\left(I‐VI\right)+{Ĥ}_{EN‐PT2}\left(VII/VIII\right)$$

where the Dyall Hamiltonian is used for the first six perturber classes. Due to the simplicity of the EN-PT zeroth order Hamiltonian, it is feasible to use the theory in its usual uncontracted form. Furthermore, the prohibitively costly four-and five-electron excitation operator terms in SC-NEVPT2 and PC-NEVPT2 are avoided entirely. Nevertheless, since the number of virtual orbitals scales with the basis set dimension, and because of the uncontracted ansatz, the second-order energy correction can involve enormous numbers of CSFs. To tame the arising computational costs, we make use of the HCI prescreening techniques that were used in the CFG-HCI method.^1^ We screen the generation of excited CFGs in the ${\mathrm{V̂}}_{a}^{\left(-1\right)}$ and ${\mathrm{V̂}}_{i}^{\left(+1\right)}$ perturber spaces based on the one-and two-electron integral and CI coefficient values

$$\left|C_{I}\right|\cdot \left|\left(pq|rs\right)\right|<T^{EN‐PT2},\mathrm{and},\left|C_{I}\right|\cdot \left|h_{pq}\right|<T^{EN‐PT2}$$ 37

Note that one of the MO indices in *h*_pq_ and (*pq*|*rs*) is either virtual or internal for ${\mathrm{V̂}}_{a}^{\left(-1\right)}$ or ${\mathrm{V̂}}_{i}^{+1}$ , respectively. The large size of the first order wave function is handled by calculating the energy correction in parallel and on-the-fly batches. This is achieved by making use of the prefix-algorithm outlined above which enables generating only batches of the |*A*⟩-configurations at a time. We refer the reader to the Supporting Information for the explicit equations and algorithmic details.

## Results

The SC-NEVPT2 and EN-SC-NEVPT2 methods were used in test calculations for (1) the N_2_ binding curve, (2) triplet-singlet gaps in polyacenes, (3) the electrocyclic ring closure reaction in cethrene and (4) methane oxidation by FeO^+^. For brevity, the SC-NEVPT2 and EN-SC-NEVPT2 methods will be referred to as NEVPT2 and ENEVPT2, respectively.

### N_2_ Binding Curve

Figure 1 presents the binding curves of the nitrogen molecule calculated with the implemented NEVPT2 and ENEVPT2 methods utilizing a valence active space of 10 electrons in 8 orbitals. The calculations were carried out in steps 0.025 Å and orbitals from the previous geometry were read in as the starting guess at the current geometry. The main focus of this set of calculations was on whether the two curves are continuous and how much the ENEVPT2 binding curve differs from that of NEVPT2.

**Figure 1:**
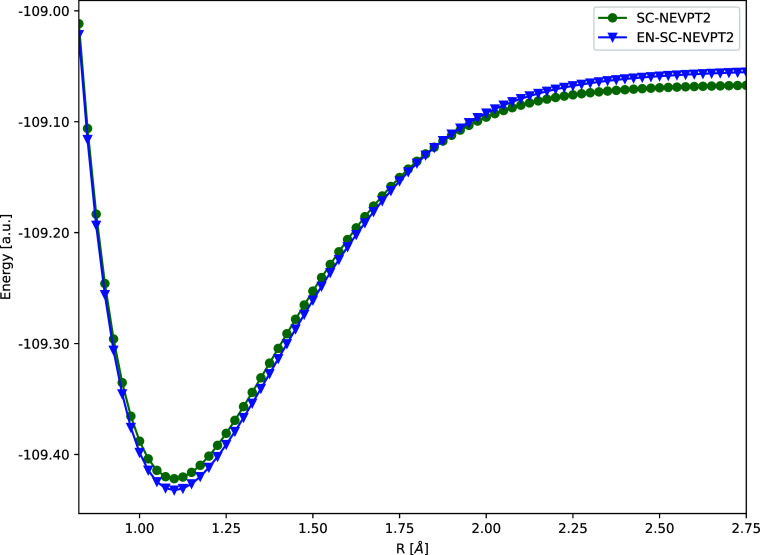
N_2_ binding curves with SC-NEVPT2 vs SC-EN-NEVPT2.

Both potential energy surfaces are smooth throughout the tested range and with both methods the equilibrium bond length is at 1.1 Å. However, discrepancies occur in the energies. With NEVPT2, the binding energy is predicted to be 222.5 kcal/mol, whereas for ENEVPT2, 236.5 kcal/mol is obtained. These values differ notably from the experimental value of around 228 kcal/mol.^51^ However, as explained in the beginning, the primary goal here is to investigate differences between the two NEVPT2 methods. Closer inspection of the potential energy surfaces reveals that at near the equilibrium and at larger distances, the energy differences occur in opposite directions. To illustrate this point, the energy difference, Δ*E* = *E*_SC‑NEVPT2_ – *E*_EN‑SC‑NEVPT2_, is shown in Figure 2. Near equilibrium, NEVPT2 yields a smaller perturbative correction compared to ENEVPT2. In contrast, near the dissociation limit, the calculated energy corrections are larger, and this trend is likely to continue after 2.75 Å. A potential reason for this result is that the EN-PT2 method is not size-consistent.^50^ Therefore, caution is warranted when using the ENEVPT2 method for comparing distant points on a potential energy surface.^52^

**Figure 2:**
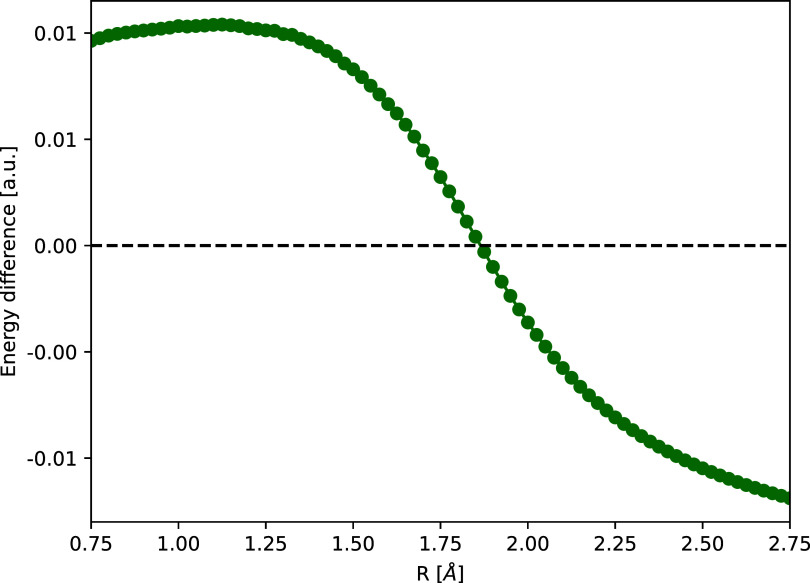
NEVPT2 vs ENEVPT2 energy differences along the N_2_ binding curve.

### Triplet-Singlet Gaps in Polyacenes

Using the NEVPT2 and ENEVPT2 methods, we calculated adiabatic singlet–triplet energy gaps, Δ*E*_*T*–*S*_ = *E*(*T*) – *E*(*S*), for polyacenes with *n* = 3 – 8 (see Figure 3). The number of active π electrons and orbitals is given by *N*_el/orb_ = 4*n* + 2, yielding active spaces from (14e, 14o) to (34e, 34o).

**Figure 3:**
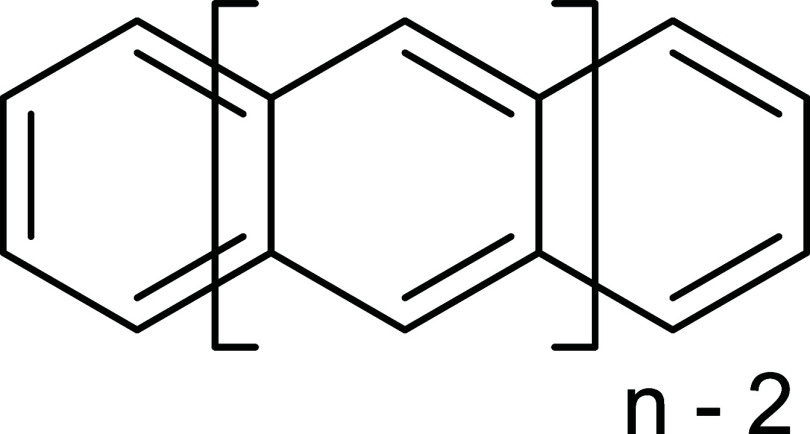
Structure of *n*-acenes for which singlet–triplet energy gaps were computed in this work.

First, we calculated the energy gaps for anthracene for which an exact CAS (14e, 14o) calculation is still feasible. Different thresholds were chosen for the reference CFG-HCI method and the steps in NEVPT2 that involve screening. For CFG-HCI, we used the following (*T*_gen_, *T*_var._) thresholds: *T*_1_^HCI^=(5 · 10^–2^,10^–4^), *T*_2_^HCI^=(1 · 10^–2^,1 · 10^–5^) and *T*_3_^HCI^=(5 · 10^–3^,1 · 10^–6^); for both the *D* ^3^ and *T* ^EN–PT2^ screening in NEVPT2 and ENEVPT2, we used: *T*_1_^NEVPT2^ = 1 · 10^–7^, *T*_2_^NEVPT2^ = 1 · 10^–8^ and *T*_3_^NEVPT2^ = 0. The calculated gaps with varying thresholds are shown in Table 1. It is evident that the chosen thresholds involved in the reference HCISCF calculation affect the results more than the NEVPT2 thresholds. Compared to *T*_2_^HCI^, the results with the tightest HCI thresholds are slightly further from the values obtained with the CAS reference. To investigate this further, we compare the calculated absolute energies to CASSCF-SC-NEVPT2 – the results are shown in Table 2.The calculated energies approach those of exact CAS upon tightening of the thresholds. However, for the triplet case, the differences are smaller. This can possibly be explained by the fact that in the CFG-HCI ansatz, all of the CSFs are taken for a given selected configuration. This can lead to an imbalance in treating different spin states where the higher spin states typically involve a larger number of CSFs.

**Table 1:** Calculated Δ*E*_*T*–*S*_ Gaps for Anthracene in Kcal/mol^a^

*T* ^HCI^/*T* ^NEVPT2^	HCISCF	ENEVPT2	NEVPT2
(1,1)	42.28	51.96	51.21
(1,2)	42.28	51.96	51.20
(1,3)	42.28	51.96	51.20
(2,1)	45.22	47.81	47.80
(2,2)	45.22	47.80	47.75
(2,3)	45.22	47.80	47.75
(3,1)	45.13	48.54	48.39
(3,2)	45.13	48.52	48.40
(3,3)	45.13	48.52	48.40
	CASSCF	SC-NEVPT2	
	45.48	47.41	

a The combinations of thresholds are defined in text. The CASSCF reference with SC-NEVPT2 is given at the bottom.

**Table 2:** Calculated NEVPT2 Energy Differences (in mHa) Compared to CASSCF-SC-NEVPT2

*T* ^HCI^/*T* ^NEVPT2^	singlet		triplet	
	ENEVPT2	NEVPT2	ENEVPT2	NEVPT2
(1,1)	–16.04	–15.44	–8.78	–9.39
(1,2)	–16.03	–15.42	–8.78	–9.38
(1,3)	–16.03	–15.42	–8.78	–9.38
(2,1)	–2.30	–4.14	–1.67	–3.52
(2,2)	–2.28	–4.00	–1.67	–3.46
(2,3)	–2.28	–4.00	–1.67	–3.46
(3,1)	–1.28	–3.01	0.51	–1.46
(3,2)	–1.25	–3.09	0.51	–1.52
(3,3)	–1.25	–3.08	0.51	–1.51

We proceed by looking at calculations on polyacenes with larger active spaces than what is feasible with the CAS method. A threshold of $T_{loose}^{D^{3}}=10^{-7}$ was used for SC-NEVPT2 and two thresholds, *T*_loose_^EN‑PT2^ = 10^–7^ and *T*_Medium_^EN‑PT2^ = 10^–8^, were tested with EN-SC-NEVPT2. The calculated vs experimental energy gaps are shown in Table 3.

**Table 3:** Calculated vs Experimental Adiabatic Triplet-Singlet Energy Gaps for N-acenes^a^

*n*	HCISCF	NEVPT2	ENEVPT2	ENEVPT2	ASCI-DSRG-	expt.^54–56^
		$\left(T_{loose}^{D^{3}}\right)$	(*T*_loose_^EN‑PT2^)	(*T*_medium_^EN‑PT2^)	MRPT2 ^53^	
3	45.00	47.83	47.83	47.82	40.2	42.6
4	34.54	31.80	32.22	32.22	27.5	29.4
5	27.18	21.30	21.72	21.74	18.7	19.8
6	22.40	14.07	14.30	14.37	12.6	12.4
7	19.54	9.09	9.23	9.36	8.4	
8	16.48	-	4.25	4.51	4.3	

a For comparison, the results with the ASCI-DSRG-MRPT2 method (without the cumulant approximation) are provided.^53^ The energies are given in kcal/mol.

With increasing number of rings the Δ*E*_*T*–*S*_ values diminish. In all cases, except for anthracene, inclusion of dynamical electron correlation results in lowering of the gaps, yielding an energy closer to experiment. Compared to the ASCI-DSRG-MRPT2 results from a previous study,^53^ the differences compared to the experiment are larger for the NEVPT2 methods. Interestingly, The ASCI-DSRG-MRPT2 method seems to slightly underestimate the gap, whereas both the NEVPT2 methods overestimate it. Overall, the calculated energy gaps are within a few kcal/mol from the experimental values with anthracene being again the only exception. In contrast to the previous section, NEVPT2 and ENEVPT2 results agree here well, although, for larger calculations, the difference between the two thresholds increases slightly for ENEVPT2. Table 4 gives the zeroth order wave function sizes and NEVPT2 timings from the calculations of the triplet state. In all cases, again except for anthracene, the triplet state involved more CSFs and hence was more expensive. The number of selected CSF ranges from about ∼8·10^4^ (n = 3) to ∼7·10^6^ (n = 8) which significantly exceeds the numbers reported for the two existing combinations of NEVPT2 and selected CI variants.^35,38^ Upon tightening the ENEVPT2 threshold 10-fold, the calculation times increased at most 2.83 times. It should be noted that the presented ENEVPT2 calculations are up to more than an order of magnitude cheaper than the corresponding NEVPT2 calculations. The most expensive ENEVPT2 calculation for the (34e, 34o) active space (n = 8) took slightly over 3 days on just one compute node with 32 CPU cores and 500GB of memory. Finally, it is noteworthy that the underlying MCSCF calculation times were negligible compared to NEVPT2. For example, one CI step in the aforementioned calculation took around 20 min.

**Table 4:** First Two Columns Contain the CSF Dimension of the Zeroth Order Wave Function for the Singlet and Triplet States, Respectively^a^,^b^

n	singlet	triplet	NEVPT2	ENEVPT2	ENEVPT2
			$\left(T_{loose}^{D^{3}}\right)$	(*T*_loose_^EN‑PT2^)	(*T*_medium_^EN‑PT2^)
3	92829	80551	6.81 · 10^2^	2.60 · 10^2^	2.46 · 10^2^
4	208428	569855	6.71 · 10^3^	9.00 · 10^2^	9.55 · 10^2^
5	710215	1923480	4.90 · 10^4^	3.92 · 10^3^	5.05 · 10^3^
6	1580235	3746000	2.70 · 10^5^	1.48 · 10^4^	2.54 · 10^4^
7	2601920	5565947	1.14 · 10^6^	4.20 · 10^4^	9.52 · 10^4^
8	3524744	7120689	-	9.75 · 10^4^	2.76 · 10^5^

a The remaining columns display NEVPT2 timings in seconds for the triplet state calculations.

b All calculations were ran on a single node with two Intel­(R) Xeon­(R) Gold 6544Y CPUs (total 32 cores) and 503 GB of memory.

#### Cethrene Ring-Closure

Here we consider the electrocyclic ring-closure reaction of cethrene.^53,57^ This system is a well-suited candidate for testing the performance of approximate CAS methods due to the involvement of the extended π-electron system. An interesting chemical aspect of the reaction is that the cyclization could proceed through two distinct pathways: conrotatory or disrotatory (see Figure 4). In case of the cethrene molecule, the reaction is believed to proceed via the conrotatory path.^53,57^ Calculated reaction free energies and barrier heights from multiconfigurational quantum chemical methods could be a valuable asset to gain insight into the observed reactivity. However, such calculations are more intricate due to the necessity of choosing an active space. Especially so in applications to reaction pathways where the active space may change significantly along the reaction coordinate.^7^ Nevertheless, we apply our implemented NEVPT2 methods here to assess their performance, with regard to both the computational resource demands and the ability to predict relative energies of structural isomers.

**Figure 4:**
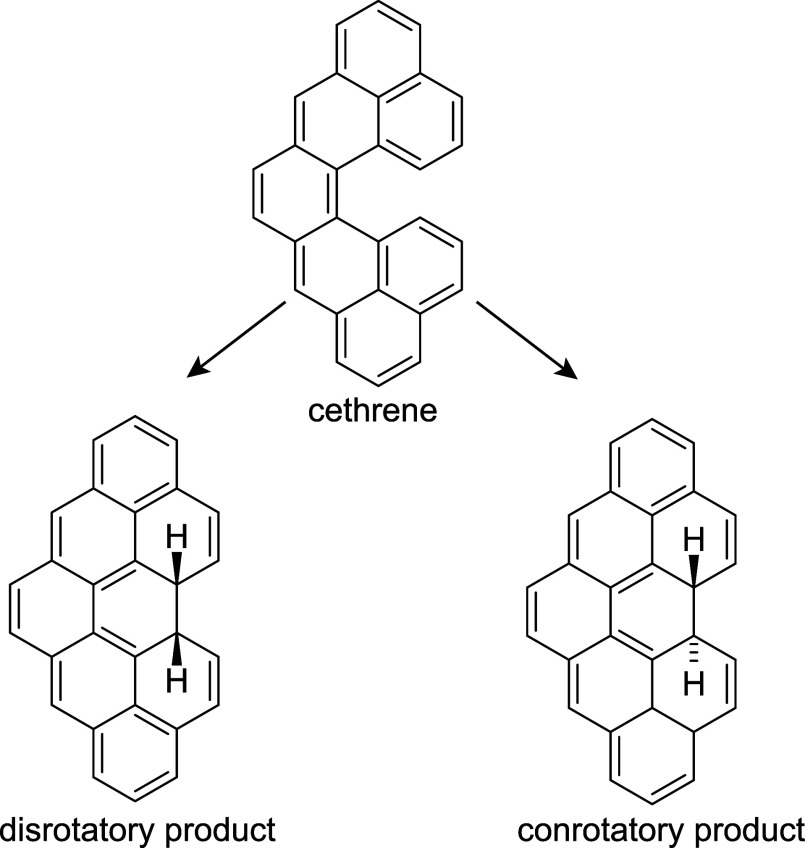
Disrotatory and conrotatory ring-closure paths of cethrene.

We carried out NEVPT2 and ENEVPT2 calculations on the reactant (R), and both, conrotatory and disrotatory, transition states as well as their corresponding products (P) – see Figures 4 and 5. All reported calculations employed active spaces consisting of all π-orbitals and electrons in the system. This yielded an active space of (28e, 28o) for the reactant and (26e, 26o) for both conrotatory and disrotatory products. However, using different active spaces along the reaction path is discouraged, since some of the orbitals would be treated at very different levels of theory. Therefore, we also carried out calculations with the (28e, 28o) active space for the products. For the transition state, the choice of a suitable active space turned out to be not entirely straightforward either. In case of the conrotatory transition state, we found that (28e, 28o) is appropriate. For the disrotatory transition state, (28e, 28o) was used as well. However, both the ASS1ST^58,59^ (active space selection based on first-order perturbation theory) calculation and the final HCISCF calculation suggested an active space of (26e, 26o) to be more appropriate based on analyzing the orbital occupation numbers. For consistency with previous studies, the results with (28e, 28o) for TS_dis._ will be shown here.

**Figure 5:**
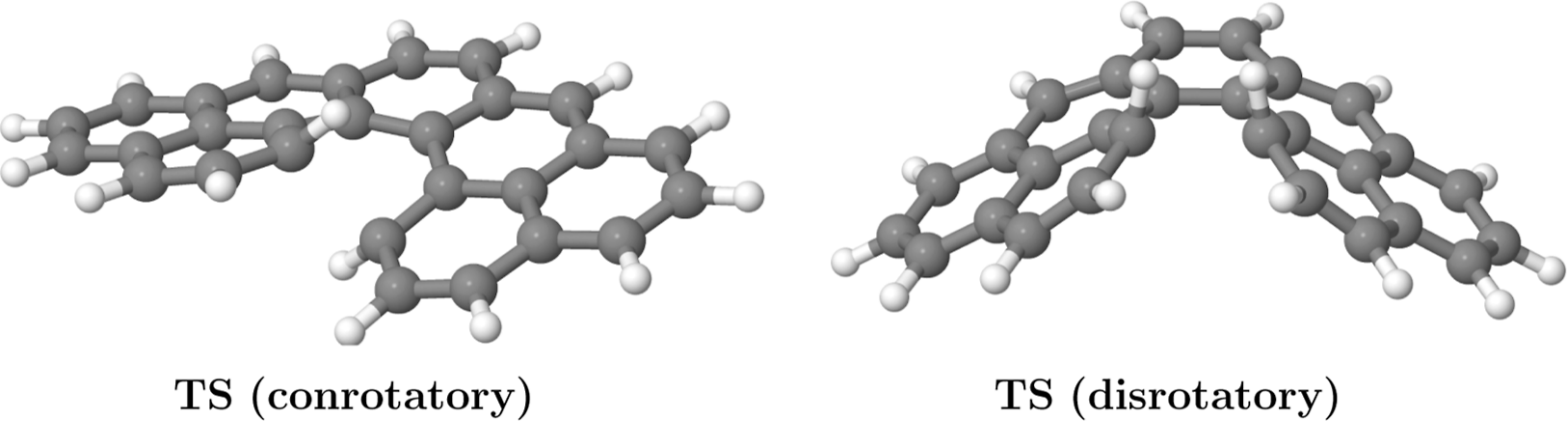
Conrotatory and disrotatory cethrene ring-closure transition states.

Table 5 shows the calculated reaction barriers and energies defined as Δ*E* ^‡^ = *E*_TS_ – *E*_R_ and Δ*E* = *E*_R_ – *E*_P_ respectively. A comparison of the NEVPT2 values with values from ENEVPT2 obtained with thresholds of *T*_loose_ = 10^–7^ and *T*_Medium_ = 10^–8^ reveals that for systems of this magnitude a tighter threshold is required to yield reliable results. The results for ENEVPT2 with medium thresholds are similar to those of regular NEVPT2 with a loose threshold. NEVPT2 calculations with a medium threshold were not carried out here due to excessively large computational demands. The results with different active space sizes differ significantly with all the methods used here. Fortunately, the interpretation of the calculated energies remains the same, favoring the conrotatory reaction path. Notably, also at the HCISCF level of theory the conrotatory pathway is already the preferred pathway. Our results agree with those of Park in predicting the conrotatory pathway to be the preferred one.^53^ Although, their barrier height of 17.1 kcal/mol calculated with ASCI-DSRG-MRPT2 is somewhat lower.

**Table 5:** Calculated Reaction Energies (Δ*E*) and Barrier Heights (Δ*E*) for Conrotatory and Disrotatory Reaction Paths of Cethrene Obtained With (28e, 28o) vs (28e, 28o) and (26e, 26o) Active Spaces^a^

pathway	HCISCF	ENEVPT2	ENEVPT2	NEVPT2
		(*T*_loose_^EN‑PT2^)	(*T*_medium_^EN‑PT2^)	$\left(T_{loose}^{D^{3}}\right)$
Δ*E* ^**‡**^ [kcal/mol]				
conrotatory (28/28)	31.61	18.50	20.34	21.14
disrotatory (28/28)	42.26	15.69	24.69	24.40
disrotatory (28/26)	56.41	27.16	26.91	23.15
Δ*E*[kcal/mol]				
conrotatory (28/28)	–18.21	8.77	–2.47	–3.78
conrotatory (28/26)	–3.83	–0.31	–15.02	–16.36
disrotatory (28/28)	–2.86	14.37	9.38	9.55
disrotatory (28/26)	11.29	0.98	–3.13	–2.95

a Active space PT2 corrections are part of the NEVPT2 energies.

Wave function sizes and NEVPT2 calculation timings corresponding to the calculations shown in Table 5 are presented in Table 6. The number of selected CSFs varies from about 3.1 million to 4.7 million, making these calculations considerably larger than what has—to our knowledge—been reported before. In all cases, the longest calculation times were needed for regular NEVPT2 calculations with loose thresholds. The longest calculation took nearly 7 days. For these calculations, the computation of three-body Dyall terms (see eq 31) constitutes the bottleneck. ENEVPT2 calculations with loose thresholds are about an order of magnitude cheaper, however, at the price of considerable loss of accuracy in this case (see above). Upon tightening the ENEVPT2 screening threshold from *T*_loose_ to *T*_Medium_ by an order of magnitude, the cost increased about 3.3 to 5.2 times. Nevertheless, the ENEVPT2 method at the *T*_Medium_ threshold remains about 2–3 times cheaper than NEVPT2 (*T*_loose_).

**Table 6:** Active Space Sizes, Numbers of CSFs in the Zeroth Order Wave Function, and Timings (seconds) for the Reported Calculations on Cethrene With SC-NEVPT2 and EN-SC-NEVPT2^a^,^b^

species	(*n*_el_, *n*_orb_)	*N*_CSF_	t_NEVPT2_ · 10^5^	t_ENEVPT2_ · 10^4^	t_ENEVPT2_ · 10^5^
			$\left(T_{loose}^{D^{3}}\right)$	(*T*_loose_^EN‑PT2^)	(*T*_medium_^EN‑PT2^)
R	28, 28	4073393	5.24	3.56	1.80
TS (con.)	28, 28	4536839	5.72	3.98	1.98
TS (dis.)	28, 28	4666648	5.16	4.14	2.16
TS (dis.)	26, 26	3412675	2.56	2.86	1.24
P (con.)	28, 28	3401245	3.93	3.76	1.68
P (con.)	26, 26	3141771	2.08	2.05	0.68
P (dis.)	28, 28	3363175	4.12	3.53	1.72
P (dis.)	26, 26	3207898	2.38	2.53	1.11

a The timings for SC-NEVPT2 correspond to loose thresholds whereas for EN-SC-NEVPT2 timings with both loose and medium thresholds are given. Abbreviations: R – reactant; TS – transition state; P – product.

b All calculations were conducted on a single node with two Intel­(R) Xeon­(R) Gold 6544Y CPUs (total 32 cores) and 503 GB of memory.

#### Methane Oxidation

As the last test case, we consider methane oxidation by FeO^+^ – see Figure 6 for the involved species. Studying this reaction pathway with multiconfigurational methods poses again an interesting challenge due to changing bonds between atoms in the course of the reaction. We carried out NEVPT2 calculations with (17e, 17o) and (21e, 21o) active spaces and ENEVPT2 calculations with an (17e, 17o) active space. In all presented NEVPT2 calculations, three thresholds were tested: *T*_loose_ = 10^–7^, *T*_Medium_ = 10^–8^ and *T*_0_ = 0. Our results indicate that in this strongly correlated case, medium thresholds might be necessary to capture all relevant correlation effects. For (21e, 21o) energy differences between the thresholds *T*_loose_ and *T*_0_ amount up to 2.4 kcal/mol. In contrast, the results were practically converged when comparing *T*_Medium_ to *T*_0_. As for ENEVPT2, the results were already converged at *T*_loose_ for the active space size considered here. Nevertheless, all of the following results discussed in this section were obtained with *T*_Medium_.

**Figure 6:**
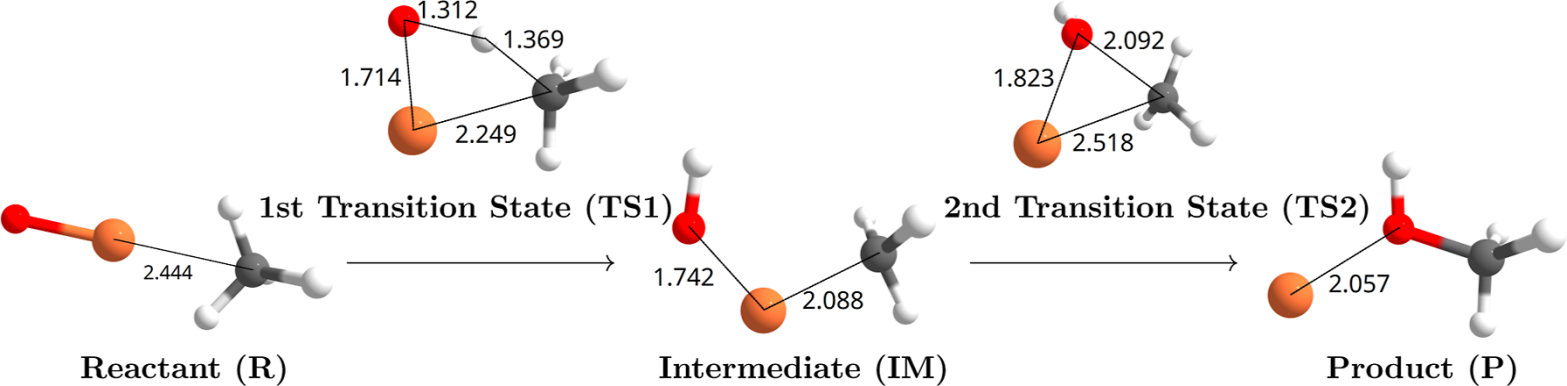
CH_4_ oxidation by FeO^+^ pathway in the quartet state. The distances are given in Å.

In Figure 7 the energy diagrams for the methane oxidation by FeO as predicted by NEVPT2 and ENEVPT2 with an active space of (17e,17o) are depicted. For this system, the calculated relative energies follow the same pattern and are quite similar although the reaction coordinate involves considerable geometrical changes. The largest calculated energy difference of 2.8 kcal/mol occurs for the intermediate state (**IM**). While regular NEVPT2 calculations were carried out also with the (21e, 21o) active space, not all corresponding ENEVPT2 calculations were successful for this active space. In some cases the calculations ran out of memory owing to the high number of unpaired electrons and the concomitant large size of coupling coefficient matrices. For example, in case of the quartet state, the number of CSFs is equal to 7072, 25194, 90440 for 17, 19, and 21 unpaired electrons, respectively. An increment of two in the number of unpaired electrons causes the coupling coefficient matrices to be over an order of magnitude larger. In case of the calculations done on, e.g., the polyacenes, where the active spaces were significantly larger, configurations with such high number of unpaired electrons were not reached in the selection process. Therefore, the performance of the presented methods depends strongly on the spin multiplicity as well as the strength of electron correlation in the system under study This brings out a prominent drawback of the presented method that is related to the CFG-based organization of the many-electron basis.^1,4^ An individual selection of CSFs would likely alleviate this issue and will therefore be implemented in due course by members of our lab.

**Figure 7:**
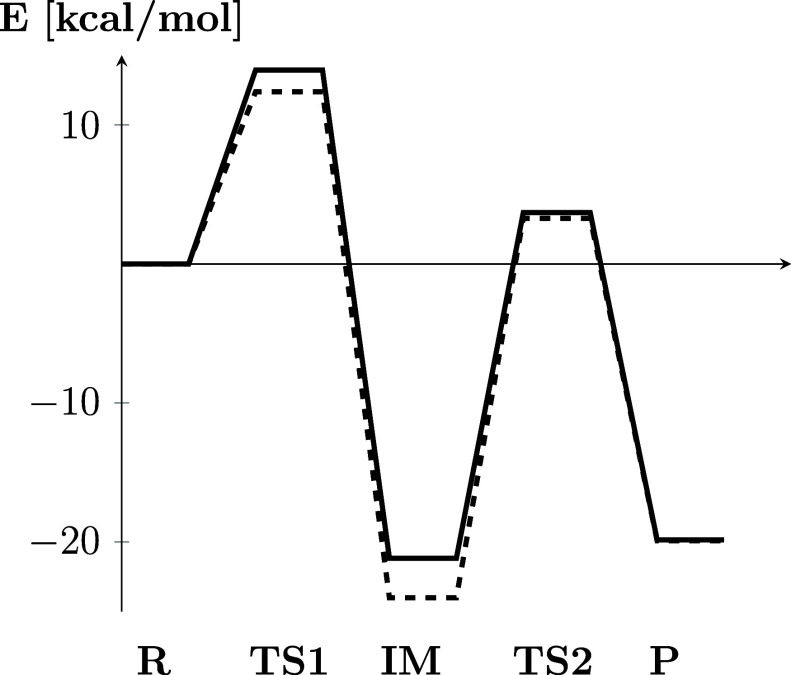
Computed relative energies of all species involed in the CH_4_ oxidation pathway. SC-NEVPT2 (solid) vs EN-SC-NEVPT2 (dashed) with (17e, 17o) active space.

A comparison of our NEVPT2 results with (21e, 21o) and (17e, 17o) active spaces is shown in Figure 8. The calculated relative energies are similar, except for the product, where a difference of 10 kcal/mol is observed. This finding could be due to an inappropriate active space or a local minimum occurring in the (17e, 17o) HCISCF calculation. The relative energies with HCISCF changed more significantly compared to NEVPT2 (see Table 7) indicating a strong dependence on the active space. Going from the (17e, 17o) active space to the (21e, 21o) active space the calculation times increased significantly from 3 to 5 h to 18 to 38 h on a single node with two Intel Xeon Gold 6544Y CPUs (32 physical cores in total).

**Figure 8:**
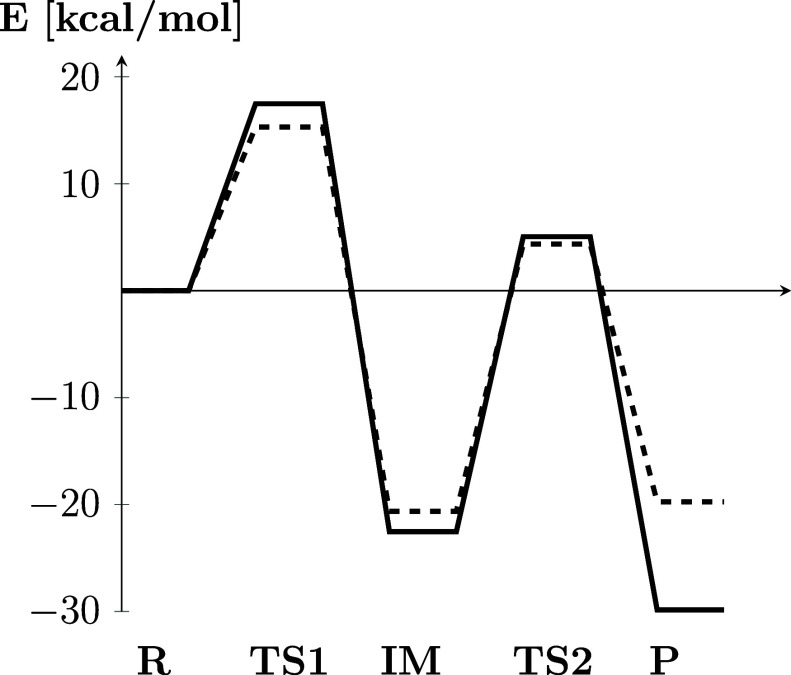
SC-NEVPT2 reaction energies for (21e, 21o - solid) and (17e, 17o—dashed) active spaces.

**Table 7:** Calculated CH_4_ Reaction Path Energy Differences (in Kcal/mol) with Respect to the Reactant (**R**)

		ASCI-DSRG-	HCISCF	SC-NEVPT2		
	**CCSD(T)** ^60^	**MRPT2** ^53^	**(17e, 17o)/(21e, 21o)**	**(17e, 17o)/(21e, 21o)**		
**TS1**	22.1	23.0	30.4	22.5	13.9	14.3
**IM**	–17.1	–13.7	–18.6	–29.2	–21.2	–23.5
**TS2**	6.6	13.4	12.0	3.6	3.7	2.6
**P**	–31.5	–31.2	–40.1	–45.7	–19.8	–30.0

We have compiled results for the calculated reaction energies from previous studies^53,60^ and compare them to the results from this study in Table 7. The final Δ*E*_R‑P_ reaction energies agree well between SC-NEVPT2 (21e, 21o) and the previous results. It is remarkable, however, that for other energies, considerable discrepancies are observed between all three studies. Besides the different wave function Ansatz, these discrepancies might originate from differences in the geometry optimization protocol (DFT vs wave function based methods), basis set size and other technical parameters. Obtaining more accurate reference energies for this system could be a worthwhile investigation in the future but is beyond the scope of this work.

## Computational Details

### N_**2**_ Binding Curve

Calculations on the nitrogen dimer were carried out with a valence orbital active space of 10 electrons in 8 orbitals and a cc-pVQZ basis set.^61^ Default thresholds of *T*_gen_ = 10^–2^ and *T*_var._ = 10^–5^ were used for HCISCF. For SC-NEVPT2 and EN-SC-NEVPT2, thresholds with the value of 10^–7^ were applied.

### Triplet-Singlet Gaps in Polyacenes

The UB3LYP/6-31G­(*d*)-optimized geometries for *n* = 3 – 7 were taken from a previous study by Schriber et al.^62^ The geometry for octacene was taken from a previous study by Park et al.^63^ For the initial starting orbitals, we used the AVAS procedure implemented in ORCA6.0.0.^64,65^ cc-pVTZ main basis set and the universal def2-JK fitting basis set were used in multireference calculations with HUMMR.^61,66^ The HCISCF calculations used the following thresholds: *T*_gen_ = 10^–2^ and *T*_var._ = 5 · 10^–5^.^1^ For SC-NEVPT2, screening threshold of *T*_loose_ = 10^–7^ was used; for EN-SC-NEVPT2 we tested both thresholds of *T*_loose_ = 10^–7^ and *T*_Medium_ = 10^–8^.

### Cethrene Ring-Closure

The HCISCF and NEVPT2 single point calculations were carried out on geometries optimized by Šolomek et al. using B3LYP with the 6–31G­(d) basis set.^57^ Starting orbitals for the final (28e28o)/(26e26o) calculations were obtained successively: (1) AVAS procedure in ORCA6.0.0 ^64,65^ (2) ASS1ST calculations with HUMMR using (12e, 12o) and (20e, 20o) active spaces.^58^ The second step turned out to be necessary due to occurrence of local minima when starting directly from AVAS orbitals. The orbitals obtained with an (20e, 20o) ASS1ST calculation were then fed to the HCISCF calculations. In the latter, thresholds of *T*_gen_ = 10^–2^ (default) and *T*_var._ = 2 · 10^–5^ were used. For SC-NEVPT2, screening threshold *T*_loose_ = 10^–7^ was used, whereas two thresholds of *T*_loose_ = 10^–7^ and *T*_Medium_ = 10^–8^ were used for EN-SC-NEVPT2. def2-TZVP main basis and universal def2-JK fitting basis sets were used in the HCISCF and NEVPT2 calculations.^66,67^

### Methane Oxidation

The quartet geometries were optimized at the ASCI-SCF (17e, 19o)/def2-SVP^67,68^ level of theory in a previous study.^53^ Based on ASS1ST calculations with the def2-SVP basis set, two kinds of active spaces were chosen: (17e, 17o) and (21e, 21o).^58^ Both SC-NEVPT2 and EN-SC-NEVPT2 calculations were carried in the (17e, 17o) active space. Only SC-NEVPT2 was run with (21e, 21o). Using the ASS1ST orbitals, HCISCF, and NEVPT2 calculations were carried out with the def2-TZVPP basis set.^67^ Default thresholds were used in HCISCF, whereas three thresholds were used in the NEVPT2 calculations: *T*_loose_ = 10^–7^, *T*_Medium_ = 10^–8^ and *T*_0_ = 0. Density fitting was applied with the universal def2-JK fitting basis set.^66^

## Conclusions

We report an implementation of two multiconfigurational perturbation theory methods based on selected CI wave functions. The first one is the strongly contracted NEVPT2 variant (SC-NEVPT2) that incorporates residual terms that vanish in the exact CAS case. In the second method, EN-SC-NEVPT2, the zeroth order Dyall Hamiltonian is mixed with the Epstein-Nesbet zeroth order Hamiltonian. Sepcifically, the EN-PT approach was used to treat the notoriously expensive ${\mathrm{V̂}}_{a}^{\left(-1\right)}$ and ${\mathrm{V̂}}_{i}^{\left(+1\right)}$ perturber classes in an uncontracted manner. As a result, the latter method does not involve calculating four- and five-electron reduced density matrices. Instead, the size of the perturber wave function there scales directly with either the virtual or internal orbital space dimension. The presented test calculations show that with increasing active space size the EN-SC-NEVPT2 method is provides significant speedups compared to the presented regular SC-NEVPT2 implementation. Although the Epstein-Nesbet perturbative energy correction is not size-consistent, both NEVPT2 methods produced similar results in most presented test calculations. The largest discrepancy was observed for the N_2_ binding curve, where EN-SC-NEVPT2 method exhibited significant overbinding.

Owing to the algorithmic details and screening, the presented methods enable MRPT calculations with up to 30 active orbitals and wave function sizes of a few million CSFs. Nevertheless, less costly methods are still desirable for even larger applications. The adiabatic connection (AC) approaches developed by Pernal et al.^69,70^ or the recently introduced Green’s functions ansatz from Wang et al.^71^ are promising candidates. Alternatively, driven similarity renormalization group (DSRG) methods have exhibited remarkable efficiency in treating dynamical electron correlation on top of large reference wave functions.^72,73^ Local formulations or tensor hypercontraction based methods might reduce the scaling of computational costs with increasing orbital space.^74–77^

## Supplementary Material



## Notes

Explicit terms and algorithms mentioned in the text (PDF)
