# Supplementary material for: An Uncontracted Epstein-Nesbet Perturbation Theory Approximation to SC-NEVPT2 Based on Spin-Pure Selected CI Wave Functions
Source: J Chem Theory Comput. 2026 Jul 8;22(14):7164–76. doi: 10.1021/acs.jctc.6c00414 (PMC13420582; doi:10.1021/acs.jctc.6c00414)
Supplement: Supplementary file 1 [file ct6c00414_si_001.pdf]

Supporting Information for "An uncontracted Epstein-Nesbet perturbation theory approximation to SC-NEVPT2 with spin-pure selected CI wave functions"

Mihkel Ugandi and Michael Roemelt*

Institut für Chemie, Humboldt-Universität zu Berlin, Brook-Taylor-Str. 2, D-12489 Berlin, Germany

E-mail: michael.roemelt@hu-berlin.de

Contents

1 SC-NEVPT2 residual terms **S2**

1.1 Explicit expressions for $D_l^{(k)}$ S3

2 EN-PT2 terms for SC-NEVPT2 **S5**

2.1 $\hat{V}_a^{(-1)}$ S5

2.2 $\hat{V}_i^{(+1)}$ S6

3 Prefixed SC-NEVPT2 and EN-SC-NEVPT2 Algorithms **S7**

3.1 Auxiliary 16 and 22 . . . . S7

3.2 1-and 2-electron Dyall Terms S9

3.3 3-electron Dyall term S11
3.4 EN-PT2 $\hat{V}_a^{(-1)}$ and $\hat{V}_i^{(+1)}$ S12

1 SC-NEVPT2 residual terms

The residual terms are defined as

$$R_l^{(k)} = E^{(0)} - \frac{1}{N_l^{(k)}} \langle 0| (V_l^{(k)})^\dagger V_l^{(k)} \hat{H}^D |0\rangle \tag{1}$$

The 0th order (approximate) CAS energy can be divided into two parts, core and active:

$$E^{(0)} = E^{(0)}_{\text{core}} + E^{(0)}_{\text{act}} \tag{2}$$

The core energy is given by

$$\begin{aligned} E^{(0)}_{\text{core}} &= 2\sum_i (h_{ii} + F^C_{ii}) \\ F^C_{pq} &= h_{pq} + \sum_i [2(ii|pq) - (ip|iq)] \quad \text{(core Fockian)} \end{aligned} \tag{3}$$

The active energy is obtained by diagonalizing the active part of the Hamiltonian,

$$\hat{H}_{\text{act}} = \sum_{tu} F^C_{tu} E_{tu} + \frac{1}{2}\sum_{tuvw} (tu|vw) e_{tu,vw} \tag{4}$$

in a space of selected CSFs, $|I\mu\rangle \in V_0$. The core energy thus merely acts as a shift on the energy eigenvalue. By letting the Dyall Hamiltonian act on a reference state, we obtain

$$\hat{H}^D |0\rangle = \hat{H}^D_{\text{inact}} |0\rangle + \hat{H}^D_{\text{act}} |0\rangle = E^{(0)}_{\text{core}} |0\rangle + \hat{H}^D_{\text{act}} |0\rangle \tag{5}$$

Plugging eq. (2) and eq. (5) into eq. (1), the residual becomes

$$\begin{aligned} R_l^{(k)} &= E_{\text{core}}^{(0)} + E_{\text{act}}^{(0)} - E_{\text{core}}^{(0)} \frac{\langle 0| (V_l^{(k)})^\dagger V_l^{(k)} |0\rangle}{N_l^{(k)}} - \frac{1}{N_l^{(k)}} \langle 0| (V_l^{(k)})^\dagger V_l^{(k)} \hat{H}_{\text{act}}^D |0\rangle \\ &= E_{\text{act}}^{(0)} - D_l^{(k)}/N_l^{(k)} \end{aligned} \tag{6}$$

By making use of the definition for the perturber norm, the core energy terms canceled and we ended up only with the active 0th order energy and the defined Dyall term,

$$D_l^{(k)} = \langle 0| (V_l^{(k)})^\dagger V_l^{(k)} \hat{H}_{\text{act}}^D |0\rangle \tag{7}$$

1.1 Explicit expressions for $D_l^{(k)}$

Later in this section are given the Dyall terms for the eight perturber classes of SC-NEVPT2. Before that, let us write some useful relations:

$$\hat{P}_0 \hat{H}_{\text{act}}^D \hat{P}_0 |0\rangle = E_{\text{act}}^{(0)} |0\rangle \tag{8}$$

$$\tilde{F}_{pq}^{\text{C}} = F_{pq}^{\text{C}} - \sum_t (tp|tq) \tag{9}$$

$$\tilde{\delta}_{pq} = 1 - \tfrac{1}{2}\delta_{pq} \tag{10}$$

The Dyall tensors that will occur are defined as

$$\Delta_{tu}^1 = \langle 0| E_{tu} H_{\text{act}}^D |0\rangle \tag{11}$$

$$\Delta_{tu,vw}^2 = \langle 0| E_{tu} E_{vw} H_{\text{act}}^D |0\rangle \tag{12}$$

$$\Delta_{tu,vw,xy}^3 = \langle 0| E_{tu} E_{vw} E_{xy} H_{\text{act}}^D |0\rangle \tag{13}$$

The Dyall term for the $\hat{V}_{ij,ab}^{(0)}$ perturber class is given by

$$D_{ij,ab}^{(0)} = 4\tilde{\delta}_{ij}\tilde{\delta}_{ab}[(ia|jb)^2 + (ib|ja)^2 - (ia|jb)(ib|ja)] \langle 0| \hat{H}_{\text{act}}^D |0\rangle \tag{14}$$

Because $\langle 0|\, \hat{H}^D_{\text{act}}\, |0\rangle = E^{(0)}_{\text{act}}$, the Dyall term becomes

$$D^{(0)}_{ij,ab} = N^{(0)}_{ij,ab} E^{(0)}_{\text{act}} \tag{15}$$

and so the residual, eq. (6), for the $\hat{V}^{(0)}_{ij,ab}$ class vanishes. For the rest of the perturber classes, the residuals don't vanish. The obtained Dyall terms for them are the following

$$D^{(-1)}_{i,ab} = \tilde{\delta}_{ab} \sum_{tt'} \{2[(ia|t'b)(ia|tb) + (ib|t'a)(ib|ta)] - (ia|t'b)(ib|ta) - (ib|t'a)(ia|tb)\}\Delta^1_{tt'} \tag{16}$$

$$\begin{aligned} D^{(+1)}_{ij,a} = \tilde{\delta}_{ij} \sum_{tt'} &\{2[(it'|ja)(it|ja) + (jt'|ia)(jt|ia)] - (it'|ja)(jt|ia) - (jt'|ia)(it|ja)\} \\ &(-\Delta^1_{t't} + 2\delta_{tt'} E^{(0)}_{\text{act}}) \end{aligned} \tag{17}$$

$$D^{(-2)}_{ab} = \tilde{\delta}_{ab} \sum_{t'u'tu} (t'b|u'a)(tb|ua)(\Delta^2_{t'u',tu} - \delta_{u't}\Delta^1_{t'u}) \tag{18}$$

$$\begin{aligned} D^{(+2)}_{ij} = \tilde{\delta}_{ij} \sum_{t'u'tu} &(iu'|jt')(iu|jt)[\Delta^2_{tu,t'u'} - \delta_{u't}\Delta^1_{t'u} + \delta_{t'u}\Delta^1_{tu'} + \delta_{u't}\Delta^1_{ut'} - 2\delta_{t't}\Delta^1_{uu'} \\ &- 2\delta_{u'u}\Delta^1_{tt'} - 2E^{(0)}_{\text{act}}(\delta_{t'u}\delta_{u't} - 2\delta_{tt'}\delta_{uu'})] \end{aligned} \tag{19}$$

$$\begin{aligned} D^{(0)}_{i,a} = \sum_{t'u'tu} &\{[2(ia|t'u')(ia|tu) - (ia|t'u')(it|ua) - (it'|u'a)(ia|tu)]\Delta^2_{u't',tu} \\ &+ (it'|u'a)(it|ua)(2\delta_{t't}\Delta^1_{u'u} - \Delta^2_{u'utt'})\} \\ &+ 2\sum_{t'u'}[2(ia|t'u') - (it'|u'a)]F^C_{ai}\Delta^1_{u't'} + 2(F^C_{ai})^2 \end{aligned} \tag{20}$$

$$\begin{aligned} D^{(-1)}_{a} = &\sum_{tuv,t'u'v'} (t'v'|u'a)(tv|ua)\Delta^3_{v't',u'u,tv} + 2\sum_{t'u'v't} (t'v'|u'a)\tilde{F}^C_{at}\Delta^2_{v't',u't} \\ &+ \sum_{t't} \tilde{F}^C_{at'}\tilde{F}^C_{at}\Delta^1_{t't} \end{aligned} \tag{21}$$

$$\begin{aligned} D^{(+1)}_{i} = &\sum_{tuv,t'u'v'} (t'v'|iu')(tv|iu)[2\delta_{u'u}\Delta^2_{v't',tv} - \Delta^3_{v't',uu',tv}] \\ &+ \sum_{t'u'v't} (t'v'|iu')F^C_{ti}[2\delta_{u't}\Delta^1_{v't'} - \Delta^2_{v't',tu'}] + \sum_{t't} F^C_{t'i}F^C_{ti}(2\delta_{t't}E^{(0)}_{\text{act}} - \Delta^1_{tt'}) \end{aligned} \tag{22}$$

2 EN-PT2 terms for SC-NEVPT2

The EN-PT2 energy correction is calculated from

$$\Delta E^{\text{EN-PT2}} = \sum_{A \in V'} \frac{(\sum_{I \in V_0} H_{AI} C_I)^2}{E^{(0)} - H_{AA}} \tag{23}$$

with the definitions

$$H_{AI} = \langle A | \hat{H} | I \rangle \quad \text{and} \quad H_{AA} = \langle A | \hat{H} | A \rangle \tag{24}$$

In here, the perturber space V' is restricted to the uncontracted $\hat{V}_a^{(-1)}$ and $\hat{V}_i^{(+1)}$ classes. For later usage, we define some matrix elements

$$\gamma_{pq}^{PQ} = \langle P | E_{pq} | Q \rangle \tag{25}$$

$$\gamma_{pq,rs}^{PQ} = \langle P | E_{pq} E_{rs} | Q \rangle \tag{26}$$

where P and Q refer to arbitrary CFGs that are connected through either single or double-excitations. In the following are given explicit expressions for the Hamiltonian matrix elements from eq. (24) in the $\hat{V}_a^{(-1)}$ and $\hat{V}_i^{(+1)}$ perturber spaces.

2.1 $\hat{V}_a^{(-1)}$

$$\begin{aligned} H_{AI} &= h'_{at} \gamma_{at}^{AI} + \sum_i (at|ii) \gamma_{at,ii}^{AI} + \tfrac{1}{2} \sum_i (it|ai) \gamma_{it,ai}^{AI} \\ &+ \tfrac{1}{2} \sum_{tuv} (at|uv) \gamma_{at,uv}^{AI} + \tfrac{1}{2} \sum_{tuv} (tu|av) \gamma_{tu,av}^{AI} \\ h'_{at} &= h_{at} - \tfrac{1}{2} \sum_i (ai|it) - \tfrac{1}{2} \sum_u (au|ut) \end{aligned} \tag{27}$$

$$
\begin{aligned}
H_{AA} &= \sum_i h'_{ii}\gamma^{AA}_{ii} + \sum_t h'_{tt}\gamma^{AA}_{tt} + h'_{aa}\gamma^{AA}_{aa} \\
&+ \tfrac{1}{2}\sum_{ij}(ii|jj)\gamma^{AA}_{ii,jj} + \sum_{it}(ii|tt)\gamma^{AA}_{ii,tt} + \sum_i (ii|aa)\gamma^{AA}_{ii,aa} \\
&+ \tfrac{1}{2}\sum_{tu}(tt|uu)\gamma^{AA}_{tt,uu} + \sum_t (tt|aa)\gamma^{AA}_{tt,aa} + \frac{1}{2}\sum_{t\neq u}(tu|ut)\gamma^{AA}_{tu,ut} \\
&+ \sum_t (at|ta)\gamma^{AA}_{at,ta} \qquad (28) \\
h'_{ii} &= h_{ii} - \tfrac{1}{2}\sum_j (ij|ji) \\
h'_{tt} &= h_{tt} - \sum_i (ti|it) - \sum_u \tfrac{1}{2}(tu|ut) \\
h'_{aa} &= h_{aa} - \sum_i (ai|ia) - \sum_t (at|ta)
\end{aligned}
$$

2.2 $\hat{V}_i^{(+1)}$

$$
\begin{aligned}
H_{AI} &= h'_{ti}\gamma^{AI}_{ti} + \tfrac{1}{2}\sum_i (tj|ii)\gamma^{AI}_{tj,ii} + \tfrac{1}{2}\sum_i (ii|tj)\gamma^{AI}_{ii,tj} \\
&+ \tfrac{1}{2}\sum_{i\neq j}(ij|ti)\gamma^{AI}_{ij,ti} + \tfrac{1}{2}\sum_{tuv}(ti|uv)\gamma^{AI}_{ti,uv} + \tfrac{1}{2}\sum_{tuv}(tu|vi)\gamma^{AI}_{tu,vi} \qquad (29) \\
h'_{ti} &= h_{ti} - \tfrac{1}{2}\sum_j (tj|ji) - \tfrac{1}{2}\sum_u (tu|ui)
\end{aligned}
$$

$$
\begin{aligned}
H_{AA} &= \sum_i h'_{ii}\gamma^{AA}_{ii} + \sum_t h'_{tt}\gamma^{AA}_{tt} + \tfrac{1}{2}\sum_{ij}(ii|jj)\gamma^{AA}_{ii,jj} \\
&+ \sum_{it}(ii|tt)\gamma^{AA}_{ii,tt} + \tfrac{1}{2}\sum_{tu}(tt|uu)\gamma^{AA}_{tt,uu} + \tfrac{1}{2}\sum_{i\neq j}(ij|ji)\gamma^{AA}_{ij,ji} \\
&+ (ti|it)\gamma^{AA}_{ti,it} + \tfrac{1}{2}\sum_{t\neq u}(tu|ut)\gamma^{AA}_{tu,ut} \qquad (30) \\
h'_{ii} &= h_{ii} - \tfrac{1}{2}\sum_j (ij|ji) \\
h'_{tt} &= h_{tt} - \sum_i (ti|it) - \tfrac{1}{2}\sum_u (tu|ut)
\end{aligned}
$$

3 Prefixed SC-NEVPT2 and EN-SC-NEVPT2 Algorithms

3.1 Auxiliary 16 and 22

The $\hat{V}_a^{(-1)}$ and $\hat{V}_i^{(+1)}$ perturber classes in SC-NEVPT2 feature terms that involve four-electron excitation operator matrix elements. Based on the original work by Angeli *et al.*, we name these terms auxiliary 16 and 22 or in short, A16 and A22. The focus here is specifically on the core-parts of the A16 and A22 terms that involve the four-electron matrix elements. The A22 term is analogous to the A16 and hence will not written explicitly. The A16 calculation involves contributions as

$$
A^{16}_{u'v't'xw'w} \leftarrow +\sum_{tuv}\sum_{IJ} C_I C_J (tu|vx) \langle I|\, E_{t'u'}E_{v'w'}E_{tu}E_{vw}\,|J\rangle \qquad (31)
$$

For a more efficient calculation, we define an intermediate,

$$
X_{K,wx} = \sum_J C_J \sum_{tuv} (tu|vx) \langle K|\, E_{tu}E_{vw}\,|J\rangle \qquad (32)
$$

The calculation of eq. (31) can then be carried out with a reduced cost,

$$A^{16}_{u'v't'xw'w} \leftarrow +\sum_{K} \langle 0| E_{t'u'} E_{v'w'} |K\rangle X_{K,wx} \tag{33}$$

where the t, u, v indices have been embedded into the X intermediate. In large calculations, the $X_{L,wx}$-intermediate can become too large to be stored in memory. The strategy here then is to form singly-and doubly-excited prefixes and form this intermediate on the fly. In testing, we found that even with the CFG prefixes, this intermediate can be too large. Therefore, an additional layer of batching was implemented where the CSF-dimension corresponding to $\{|L\rangle\}$ was limited to one million. The resulting algorithm is given in algorithm 1.

Algorithm 1: Hybrid OMP/MPI-parallel core-A16/A22 build

1: Gather prefixes present in V_0, make doubly excited CFG prefixes, $\{|I_P\rangle\} \xrightarrow{E_{tu}E_{vw}} \{|K_P\rangle\}$, and form the union, $\Pi = \{|I_P\rangle\} \cup \{|K_P\rangle\}$. Split the list of prefixes, Π, among parallel MPI processes.

2: Form prefix batches, $\{B\}$, with the batch size equal or less than the number of parallel OMP threads.

3: Loop over the prefix batches, B, in serial.

- 4: Complete excited RI CFGs from the prefixes, $\{|K_P\rangle\} \rightarrow |K\rangle$. Each OMP thread takes at most one prefix, $|K_P\rangle$, at a time.
- 5: Turn the RI CFGs into batches with a maximal size (10^6 CSFs).
- 6: Loop over the RI CFG batches in serial.
  - 7: Loop over the RI CFGs in a round-robin OMP parallelized fashion, create the two-electron connections, and build the $X_{L,wx}$ intermediate.
  - 8: Calculate the necessary coupling coefficients, A^{IJ}_{tu}.
  - 9: Loop over the indices (t', u', v', w') with cyclic distribution over OMP threads. Each OMP thread takes at most one tuple at a time.
    - 10: Form the two-electron connections, $\langle 0| \xrightarrow{E_{tu}E_{vw}} \langle K|$, and calculate the contributions to A16.

11: Parallel-reduce $A^{16,\text{core}}_{u'v't'xw'w}$ across MPI processes.

3.2 1-and 2-electron Dyall Terms

Using the configurational RI expansion, the one-and two electron core Dyall terms can be written as

$$\Delta^1_{tu} = \sum_K \langle 0| E_{tu} |K\rangle \langle K| \hat{H}^D_{\text{act}} |0\rangle \tag{34}$$

$$\Delta^2_{tuvw} = \sum_K \langle 0| E_{tu}E_{vw} |K\rangle \langle K| \hat{H}^D_{\text{act}} |0\rangle \tag{35}$$

Defining the sigma term, $\sigma_K = \langle K | \hat{H}^D_{\text{act}} | 0 \rangle$, the Dyall terms can be calculated as

$$\Delta^1_{tu} = \sum_K \langle 0 | E_{tu} | K \rangle \, \sigma_K \tag{36}$$

$$\Delta^2_{tuvw} = \sum_K \langle 0 | E_{tu} E_{vw} | K \rangle \, \sigma_K \tag{37}$$

The algorithmic steps are provided in Algorithm 2. We note that compared to the 3PDM and A16/A22 builds, evaluation of the one-and two-electron Dyall terms is considerably simpler. Unlike in the previous cases, the target quantities have fewer indices and are therefore feasible to keep locally in memory by each parallel thread. The indices of the Dyall Hamiltonian are buried in the sigma term and hence, a low-rank intermediate can be calculated for each prefix on the fly. Thus, the presence of the one-and two-electron residuals does not usually cause performance bottlenecks.

Algorithm 2: Hybrid OMP/MPI-parallel core D^1 and D^2 build

1: Gather prefixes present in V_0, make doubly excited CFG prefixes, $\{|I_P\rangle\} \xrightarrow{E_{tu}E_{vw}} \{|K_P\rangle\}$, and form the union, $\Pi = \{|I_P\rangle\} \cup \{|K_P\rangle\}$. Split the list of prefixes, Π, among parallel MPI processes.

2: Loop over the prefixes, $|K_P\rangle$, with a cyclic distribution over OMP threads.

  3: Complete the RI space CFGs, $\{|K_P\rangle\} \xrightarrow{E_{tu}E_{vw}} \{|K\rangle\}$.

  4: Find the one-and two-electron connections from $|0\rangle$ to the RI space, $|K\rangle$.

  5: Calculate the necessary coupling coefficients, $A^{IJ}_{tu} = \langle I | E_{tu} | J \rangle$.

  6: Calculate the sigma term, $\sigma_K = \langle K | \hat{H}^D_{\text{act}} | 0 \rangle$.

  7: Calculate contributions to Δ^1_{tu} and Δ^2_{tuvw},

$$\Delta^1_{tu} = \sum_K \langle 0 | E_{tu} | K \rangle \, \sigma_K \qquad \Delta^2_{tuvw} = \sum_K \langle 0 | E_{tu} E_{vw} | K \rangle \, \sigma_K$$

8: Parallel-reduce Δ^1_{tu} and Δ^2_{tuvw} across OMP threads.

9: Parallel-reduce Δ^1_{tu} and Δ^2_{tuvw} across MPI processes.

3.3 3-electron Dyall term

The 3-electron Dyall term can be calculated in an analogous manner to the one-and two-electron terms in the previous section,

$$\Delta^3_{tuvwxy} = \sum_K \langle 0| E_{tu}E_{vw}E_{xy} |K\rangle \langle K| \hat{H}^D_{\text{act}} |0\rangle \tag{38}$$

However, the presence of three-electron connections makes the calculation significantly more costly. We apply screening of the three-electron connections using CI coefficients and the sigma-term,

$$\max_\mu |C_{I\mu}| \cdot \max_\nu |\sigma_{K\nu}| < T_{\Delta^3} \tag{39}$$

The default threshold is $T_{\Delta^3} = 10^{-8}$ in the HUMMR program, but this value can be controlled by the user. The final Δ^3_{tuvwxy} algorithm is given in Algorithm 3. Using an analogous but simpler algorithm, the coupling coefficients are pre-calculated here as well.

Algorithm 3: Hybrid OMP/MPI-parallel Δ^3 build

1: Gather prefixes present in V_0, make doubly excited CFG prefixes, $\{|I_P\rangle\} \xrightarrow{E_{tu}E_{vw}} \{|K_P\rangle\}$, and form the union, $\Pi = \{|I_P\rangle\} \cup \{|K_P\rangle\}$. Split the list of prefixes, Π, among parallel MPI processes.

2: Form prefix batches, $\{B\}$, with the batch size equal or less than the number of parallel OMP threads.

3: Loop over the prefix batches, B, in serial.

  4: Complete excited RI CFGs from the prefixes, $\{|K_P\rangle\} \rightarrow \{|K\rangle\}$ Each OMP thread takes at most one prefix at a time.

  5: Calculate the sigma term, $\sigma_K = \langle K| \hat{H}^D_{\text{act}} |0\rangle$.

  6: Loop over the index tuples, (t, u, v, w, x, y), using cyclic distribution over OMP threads.

    7: Form three-electron connections from the RI space to the reference space, $|K\rangle \xrightarrow{E_{tu}E_{vw}E_{xy}} |0\rangle$. Screening from eq. (39) is applied here.

    8: Calculate contributions to the three-electron Dyall term,

$$\langle 0| E_{tu}E_{vw}E_{xy} |K\rangle \, \sigma_K \rightarrow \Delta^3_{tuvwxy} \tag{40}$$

  9: Parallel-reduce Δ^3_{tuvwxy} across MPI processes.

3.4 EN-PT2 $\hat{V}_a^{(-1)}$ and $\hat{V}_i^{(+1)}$

In the EN-SC-NEVPT2 method the orbital occupations can differ by one in the internal or external space for the $\hat{V}_i^{(+1)}$ or $\hat{V}^{(+1)}$ perturber class, respectively. The prefixes are generated by considering only the active orbital space and the internal or external orbitals are handled on the fly. For the $\hat{V}_i^{(+1)}$ class, there can be up to two creations and one annihilation in the active space, whereas for the $\hat{V}_a^{(-1)}$ class, there can be up to two annihilations one creation.

The second-order energy is calculated from the general formula

$$\Delta E^{\text{EN-PT2}} = \sum_{A \in V'} \frac{\sigma_A^2}{E^{(0)} - H_{AA}} \tag{41}$$

where the sigma terms is defined as

$$\sigma_A = \sum_{I \in V_0} \langle A| \hat{H} |I\rangle C_I \tag{42}$$

and the Hamiltonian diagonal in the perturber space is $H_{AA} = \langle A| \hat{H} |A\rangle$. The final algorithm for calculating this energy correction is analogous for $\hat{V}_a^{(-1)}$ and $\hat{V}_i^{(+1)}$. Hence, we shall provide an example of the algorithm for only the $\hat{V}_a^{(-1)}$ class – see Algorithm 4.

Algorithm 4: Hybrid OMP/MPI-parallel calculation of $E^{(2)}$ for $\hat{V}_a^{-1}$

1: Gather prefixes present in V_0, make excited prefixes with one creation and two annihilations, $\{|I_P\rangle\} \xrightarrow{\hat{a}_t^\dagger \hat{a}_u \hat{a}_v} \{|K_P\rangle\}$, and form the union, $\Pi = \{|I_P\rangle \cup |K_P\rangle\}$. Split the list of prefixes, Π, among parallel MPI processes.

2: Loop over the prefixes K_P with a cyclic distribution over OMP threads. Each thread gets at most one prefix at a time.

  3: Create the perturber space wave function and the configuration connections.

  4: Make diagonal connections, $\langle A| E_{tu}E_{ut} |A\rangle$.

  5: Calculate the one-electron coupling coefficients, A_{tu}^{IJ}.

  6: Calculate the sigma and diagonal terms, σ_A and H_{AA}.

  7: Calculate a contribution to the energy correction given in eq. (41).

8: Parallel-reduce the energy $E^{(2)}$ across OMP threads.

9: Parallel-reduce the energy $E^{(2)}$ across MPI processes.
